# The effects of different lookback periods on the sociodemographic structure of the study population and on the estimation of incidence rates: analyses with German claims data

**DOI:** 10.1186/s12874-020-01108-6

**Published:** 2020-09-11

**Authors:** Jelena Epping, Siegfried Geyer, Juliane Tetzlaff

**Affiliations:** Medical Sociology Unit, Hannover Medical School, Carl-Neuberg-Str 1, 30625 Hannover, Germany

**Keywords:** Lookback period, Pre-observation, Wash-out period, Claims data, Selectivity, Incidence, Socioeconomic status, Age structure, Social gradient

## Abstract

**Background:**

Defining incident cases has always been a challenging issue for researchers working with routine data. Lookback periods should enable researchers to identify and exclude recurrent cases and increase the accuracy of the incidence estimation. There are different recommendations for lookback periods depending on a disease entity of up to 10 years. Well-known drawbacks of the application of lookback periods are shorter remaining observation period in the dataset or smaller number of cases. The problem of selectivity of the remaining population after introducing lookback periods has not been considered in the literature until now.

**Methods:**

The analyses were performed with pseudonymized claims data of a German statutory health insurance fund with annual case numbers of about 2,1 million insured persons. Proportions of study population excluded due to the application of lookback periods are shown according to age, occupational qualification and income. Myocardial infarction and stroke were used to demonstrate changes in incidence rates after applying lookback periods of up to 5 years.

**Results:**

Younger individuals show substantial dropouts after the application of lookback periods. Furthermore, there are selectivities regarding occupational qualification and income, which cannot be handled by age standardization. Due to selective dropouts of younger individuals, crude incidence rates of myocardial infarction and stroke increase after applying lookback periods. Depending on the income group, age-standardized incidence rates changed differentially, leading to a decrease and possible underestimation of the social gradient after applying lookback periods.

**Conclusions:**

Selectivity analyses regarding age and sociodemographic structure should be performed for the study population after applying lookback periods since the selectivity can affect the outcome especially in health care research. The selectivity effects might occur not only in claims data of one health insurance fund, but also in other longitudinal data with left- or right-censoring not covering the whole population. The effects may also apply to health care systems with a mix of public and private health insurance. A trade-off has to be considered between selectivity effects and eliminating recurrent events for more accuracy in the definition of incidence.

## Background

Incidence is one of the key indicators in epidemiologic and public health research. Incidence rate is defined as “the occurrence of new cases of disease per unit of person-time” [1] where both the definition of “new cases” as well as the decision about the denominator can be challenging issues for a researcher and require careful considerations. For the definition of new cases in survey studies, researchers rely on the accurate recalls of respondents that can be more or less precise depending on the disease [2–4]. For administrative data strategies have to be found for differentiating between incident and prevalent cases. In this paper, incident cases are defined as first ever occurrences of a disease. One of the most popular solutions is the definition of a disease-free pre-observation period, also called lookback period, in order to distinguish between prevalent or recurrent and incident cases. Possible consequences of the application of lookback periods for the sociodemographic structure of the study population have not been analyzed until now.

Especially for studies on morbidity compression or morbidity expansion it is crucial to make the best possible estimation of the first ever morbid event in a lifetime. This may be particularly challenging since the underlying databases usually contain left- and right-censoring. Therefore, lifetime incidence rates usually are based on the best possible approximation. Furthermore, reliable incidence estimates are critical to public health experts in need of accurate assessments to effectively plan health care provision [5].

The length of lookback periods (also known as clearance periods, pre-observation time or washout periods) may vary between studies and diseases. Some recommendations regarding the length of lookback periods have been published for cancer (at least 2 years, [6]), pharmacoepidemiological studies (longer than 12 months, [7]), diabetes (10 years, [8]) and stroke (10 years, [9]). For myocardial infarction (MI), a “standard” of 1 year seems to be common [6] as several studies show, that the number of recurrent events within 1 year after the first MI amounted to the half [10] or even 63% [11] of all recurrent events occurred within the observation period of 7 years [10] or 12 years [11].

The consequences of applying different lookback periods have been investigated in terms of estimating incident rates of cancer [6], stroke [9], myocardial infarction [12] and chronic conditions [5]. Czwikla and colleagues calculated cancer incidence rates in German claims data and could almost fully eliminate the existing differences to the benchmark data of the German cancer registry after the application of 7 years of lookback period [6]. In Australian hospital data, Worthington and colleagues detected 14% of stroke cases as prevalent after extending the lookback period up to 12 years [9], though no quantification of the incidence rate was performed. For myocardial infarction, incidence rate ratios were analyzed in a Norwegian study by Sulo and colleagues [12] after applying up to 10 years of lookback period. As a result, incidence rates declined by 4,2% for men and by 7,3% for women due to the application of a lookback period of 10 years [12]. In a further study by Rassen and colleagues, several chronic conditions in UK and US electronic health records were used as examples for demonstrating the effects of lookback periods on the estimation of prevalence and incidence rates [5]. The resulting variations in prevalence and incidence estimates were impressive and lead to the discussion about the necessity of more transparency in defining the cases and the population at risk [5].

To the best of our knowledge, potentially underlying shifts in the sociodemographic structure of a study population as a consequence of applying a lookback period have not been discussed so far. Such changes could be expected in studies with claims data from health insurance funds (HIF). In Germany numerous HIF are available and transitions between them are possible or sometimes even necessary e.g., due to changing an employer. At the same time, the vast majority of studies with claims data in Germany are conducted with data from one HIF. In such datasets no information is available for the time period before the entry date, leading to left-censoring in the data. On the other hand, for individuals leaving the HIF right-censoring occurs, since no information is available for the remaining observation time. If a lookback period is applied, the insured persons leaving the HIF or entering it during the lookback period would be excluded. Sociodemographic characteristics of persons changing their HIF during the lookback period of a study can differ from the remaining population [13] and thus can affect the composition of the study population, i.e. the denominator for the calculation of incidence rates. These effects can be expected also in health care systems with a mix of private and public insurance e.g., in the USA, but also in other longitudinal databases with censored data.

The purpose of this study is on the one hand to describe changes of the sociodemographic structure of a HIF population in Lower Saxony, Germany, after applying different lookback periods of one, three and five years. Changes will be examined in terms of age structure, occupational qualification and income. At the second step of the analyses we quantify changes in incidence rates of myocardial infarction and stroke after applying lookback periods of up to 5 years. Due to the occurrence of both disease entities in older ages [14, 15] and the presence of social gradient in both [16, 17], we expect to observe changes in incidence rates.

## Methods

The following analyses were performed with anonymized data of a statutory health insurance (SHI) fund Allgemeine Ortskrankenkasse Niedersachsen (AOKN) in Germany, covering over 37% of the residents of the federal state Lower Saxony [18]. Since 2009 health insurance is compulsory for all German residents, and in 2015 only 0.1% were uninsured [19]. Below a certain income threshold, health insurance within the statutory system is mandatory, and in 2015 this applied to 88% of all permanent residents [19]. Within the statutory health care system, the amount of health care coverage is the same for all insured individuals, thus it is not comparable with a health plan, which is usually based on individual contracts between the insured individual and the insurance company. Claims data from SHI depict health care activities fairly complete as all payments from insurers to providers are registered [14]. Comparative analyses have shown that the distributions of age and sex of the analyzed insurance population do not differ from those of Lower Saxony and Germany. Though, the insurance population had a higher proportion of individuals with lower occupational qualifications [20]. This implies that incidence rates based on the insurance population are probably higher compared to the nationwide level.

The dataset used for this analysis comprised about 2,1 million insured persons aged 25 years and older. The dataset includes in-patient data with diagnoses coded according to ICD-10-GM [21], as well as extensive information on the insurance history with date specifications on entrance or leave as well as socio-demographic information like birth year, sex, occupational qualification and income. The analyses were performed with STATA MP 15.0.

### Transition between SHI funds

In Germany there are 117 SHI funds, 88 of which are open for all residents with some regional restrictions [22]. After joining one SHI fund, a person is bound for 18 months to this fund [23]. Although the amount of health care coverage is set by law [23] and is fairly the same for all individuals insured within the SHI system, there are extended benefits some SHI funds offer in order to attract new members. For example, costs for osteopathy or homeopathy can be refunded by AOKN up to a maximum of 250 Euro per year [24] even though these medical services are not part of the health care catalogue guaranteed by law [23]. Introducing such extended services can lead to increased movements between SHI funds.

Changing an SHI fund can also be motivated by changing the place of residence or occupation. The latter case can implicate entering a health insurance fund of the employer. Furthermore non-working spouses are co-insured with their working spouse if he or she is insured within an SHI fund. Thus, a change of an SHI fund can also be caused by a job loss, which is accompanied by a co-insurance with the working spouse in another SHI fund. In 2016, about 2% of the individuals insured within the SHI system changed their health insurance fund [25].

### Analysis part 1: comparison of the sociodemographic structure

Changes of the sociodemographic structure of the insurance population in 2017, which occurred due to the application of different lookback periods, were analyzed with respect to age, occupational qualification and income. For the analysis of these changes four datasets (subpopulations) were generated: 1) individuals insured at least for 1 day in 2017 (BASE); 2) individuals insured throughout the year 2016 and at least 1 day in 2017 (CON1 for at least 1 year of continuous insurance); 3) individuals insured continuously during 2014 till 2016 and at least 1 day in 2017 (CON3), and 4) individuals insured continuously during 2012 till 2016 and at least 1 day in 2017 (CON5).

We compared the age structure of the generated subpopulations in 5-year age intervals (25–29, 30–34 etc.). Furthermore, we were interested in the selectivity of dropouts regarding occupational qualification. In the German social security system, employers have the obligation to report yearly on qualification, occupation, salary, status as temporary worker and status as full- or part-time employee for all their employees [26]. German statutory health insurance funds are obliged to gather this information and to report it to the federal institutions [26]. Hence, claims data of SHI funds contain information on qualification for insured persons with at least one employment episode during their insurance history. Occupational qualification is coded in seven groups: (1) without vocational training, (2) with vocational training, (3) master craftsman, (4) bachelor degree, (5) diploma or master degree, and (6) PhD. The last group (7) includes employees whose qualification level was coded as unknown, which relates mostly to persons with a foreign degree or in temporary jobs.

Income is the third dimension we chose for analyzing the selectivity of dropouts. Income data are available for employees (individual salary up to the social security contribution ceiling of 76,200€ [23]) as well as for pensioners (individual pension up to the ceiling of 34,437 € [27]). For our analysis, income is classified into three groups in relation to the nationwide average income in Germany as published by the Federal Statistical Office [28]. The low income group comprises pre-tax annual income of < 40% of the average income in Germany (< 14′000 €), the high income group lies above 80% of the average income in Germany (more than 29′000 €), and the middle group is in between. The fourth group consists of persons without information on individual income, such as students, unemployed, housewives and househusbands, and similar.

### Analysis part 2: changes in incidence rates

Age-specific incidence rates of myocardial infarction (MI) and stroke were calculated for the year 2017 using the different subpopulations BASE, CON1, CON3 and CON5, as described above. At the second step, age-specific incidence rates were calculated stratified for gender and income groups. Thus possible changes in the social gradient of MI and stroke can be shown, if incidence rates shift differentially in each income group due to the application of lookback periods. Age-standardization at the BASE-population was performed in order to avoid a commingling of age and SES-effects.

At the first step, persons with an MI (I21 according to ICD-10-GM) or a stroke diagnosis (I60-I64) in the year 2017 were identified (subpopulation BASE). For this calculation the main diagnosis in the in-patient data was used, since the first treatment usually takes place in a hospital.

At the second step the subpopulation with 1 year lookback period was used (CON1). The MI- and stroke cases in 2017 were adjusted for persons, who had experienced an MI or a stroke event in the year 2016 (separate calculation for stroke and MI). The adjustment was performed for both the numerator and the denominator, since individuals with an experienced event in 2016 are not any more at risk of undergoing an incident MI or stroke event in 2017. The procedure was repeated for the other subpopulations CON 3 (lookback period 2014–2016), and CON5 (lookback period 2012–2016), resulting in adjusted incidence rates for 3 and 5 years of lookback periods.

Furthermore, a specific correction could be made for MI cases with the ICD-code I25.2: Earlier cases of MI were excluded if they had been documented during a hospital stay as a secondary diagnosis (concomitant disease).

## Results

Altogether 2,060,395 individuals above 24 years of age were insured by the AOKN in 2017 (51.3% were women). Out of them 1,830,100 constitute the subpopulation CON1 with the precondition of one year lookback period (− 11.2%). 1,710,781 individuals were insured throughout 2014 until 2016 and for at least 1 day in 2017 (CON3; − 6.5%). Finally, 1,623,184 met the condition of being insured for the time period 2012 to 2016 and for at least 1 day in 2017 (CON5; − 5.1%). About 93% of the BASE-subpopulation was insured the entire year 2017; for all other subpopulations the proportion of continuously insured individuals throughout the year 2017 was 99%.

A brief description of the sociodemographic characteristics of the BASE-subpopulation can be found in Table 1. In the next section, we present the proportions of excluded individuals due to the application of different lookback periods according to age structure, occupational qualification and income.

**Table 1:** Sociodemographic characteristics of persons of 25 years of age and above insured in 2017 without any preconditions (BASE subpopulation)

	Male		Female	
25–34 years	210,541	21.0%	184,730	17.5%
35–44 years	169,466	16.9%	153,846	14.6%
45–54 years	210,458	21.0%	197,943	18.7%
55–64 years	178,316	17.8%	176,663	16.7%
65–74 years	112,623	11.2%	126,146	11.9%
75–84 years	95,961	9.6%	145,101	13.7%
85+ years	26,696	2.7%	71,905	6.8%
Total	1,004,061	100.0%	1,056,334	100.0%
Qualification^a^				
without vocational training	73,708	12.3%	54,278	12.1%
with vocational training	332,122	55.3%	265,326	59.3%
master craftsman	23,390	3.9%	8280	1.9%
bachelor	7143	1.2%	7177	1.6%
diploma or master degree	19,090	3.2%	21,549	4.8%
PhD	1300	0.2%	1047	0.2%
unknown	143,795	23.9%	89,488	20.0%
Total	600,548	100.0%	447,145	100.0%
Income				
low (≤40% of the AIG^b^)	108,434	10.8%	287,003	27.2%
middle (> 40% to ≤80% of the AIG^b^	283,915	28.3%	289,089	27.4%
high (> 80% of the AIG^b^)	293,605	29.2%	107,344	10.2%
unknown	318,107	31.7%	372,898	35.3%
Total	1,004,061	100.0%	1,056,334	100.0%
Myocardial infarction	4056		2317	
Stroke	4705		4777	

^a^ - Only persons with at least one employment episode are included for the depiction of the occupational qualification

^b^– AIG: average income in Germany as published by the Federal Statistical Office

**Fig. 1:**
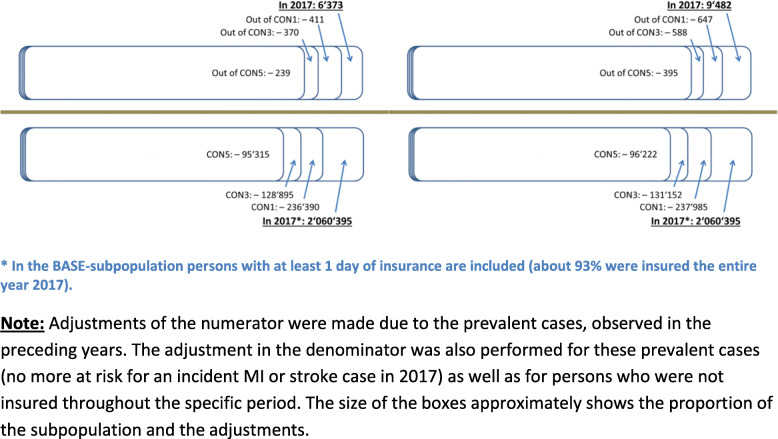
Size of the subpopulations for myocardial infarction (left panel) and stroke (right panel), divided into the numerator (number of MI- and stroke-cases; upper panels) and denominator (applied subpopulations; lower panels)

In the second part, we describe changes of incidence rates for 2017 after application of different lookback periods of one, three or five years, stratified by sex and income group. In order to control for possible differences of age structure between income groups, incidence rates are calculated age-standardized within each subpopulation. The size of each subpopulation for incidence calculations is shown in Fig. 1.

### Comparison of subpopulations based on different lookback periods

At the first step we compared the generated subpopulations by age, occupational qualification and income. The subpopulation without preconditions (BASE) was matched with each CON-subpopulation separately.

**Fig. 2:**
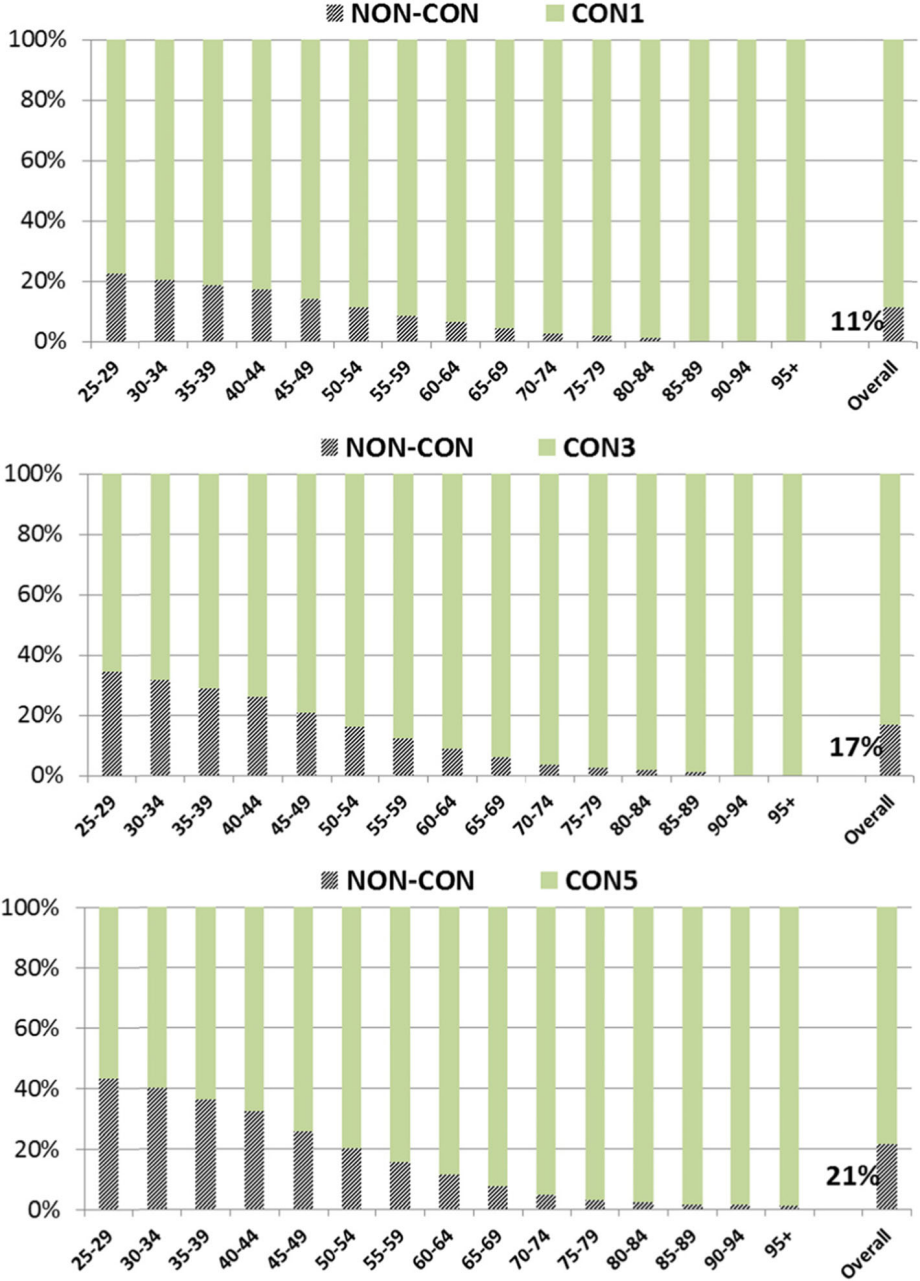
Structure of the subpopulation with one (CON1, upper panel), three (CON3, middle panel) or five (CON5, lower panel) years of continuous insurance (light green), stratified by age groups, after matching to the insured individuals without the respective precondition (striped)

The following Fig. 2 shows the proportion of insured individuals with a respective precondition of one, three or five years of continuous insurance in light green color. Striped bar sections show the proportion of individuals who do not meet the precondition of continuous insurance. In total, 11% of the population did not meet the precondition of one year of continuous insurance (male versus female: 12% vs. 10%), 17% in case of 3 years (19% vs. 15%) and 21% in case of 5 years (24% vs. 19%).

Among younger insured persons below age 45 between 17 and 22% of the subpopulation are excluded after the precondition of 1 year lookback period was applied (CON1), between 26 and 35% in the CON3- and between 32 and 43% in the CON5-subpopulation (Fig. 2). There were slight differences between men and women regarding the exclusion of individuals in younger age groups with higher proportions of excluded men (e.g. 34% vs. 30% in women in the age group 40–44 years for the CON5-subpopulation).

On the other hand, there are quite small proportions of excluded individuals aged 75 years and older ranging from 0,3% for those aged 95 and older in the CON1- subpopulation up to 3% for individuals aged 75–79 years in the CON5. Thus, the age structure of the population is changing towards older insured persons after the application of lookback periods. There were no differences in exclusion proportions between men and women in the higher age groups. As a result of the application of lookback periods, the median age shifted from 51 to 54 years in men (interquartile range 37–63 years before to 41–67 years afterwards) and from 54 to 58 years in women (40–70 years to 44–74 years, respectively).

**Fig. 3:**
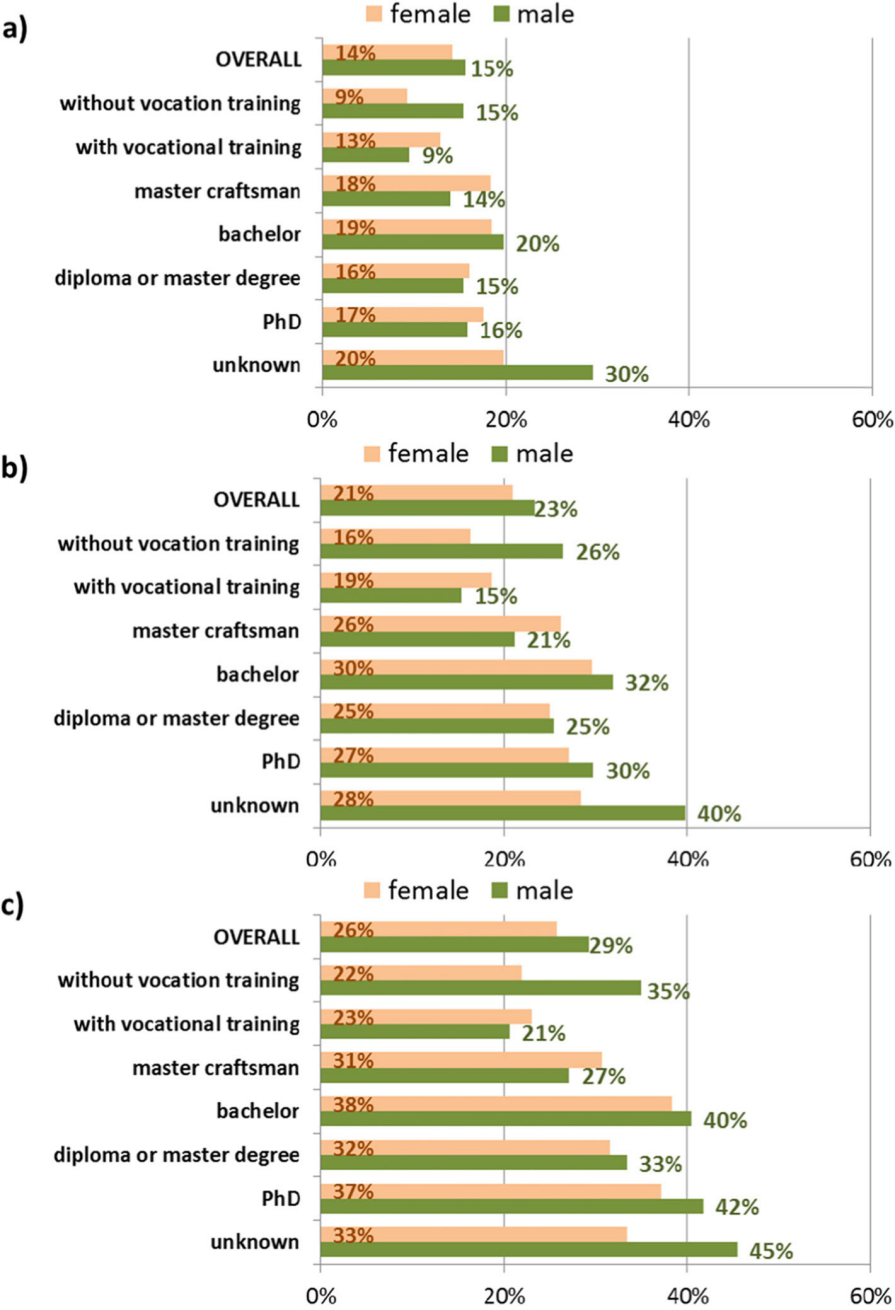
Proportions of excluded individuals by occupational qualification after application of one (CON1: a), three (CON3: b) and five (CON5: c) years of lookback period, stratified by sex, based on the population with at least one employment episode

The second attribute for identifying the selectivity due to the application of lookback periods is the occupational qualification of the insured individuals (Fig. 3). As stated in “Methods and Data” the information on occupational qualification is only available for individuals who have had at least one employment episode during their insurance history at AOKN. Thus, 1047,693 individuals could be included in the next analysis step (42.6% women). Since men and women differ in proportions of excluded individuals after the application of lookback periods, all results are stratified by sex.

In total, 15% of individuals with an employment episode have been excluded from the study population after applying a lookback period of 1 year. This proportion increased to 22% for the CON3-subpopulation and to 28% for the CON5-subpopulation with only slight differences between men and women.

In all three matches the group of individuals without vocational training and persons with a bachelor degree showed higher proportions of excluded persons than the average of the study population. This applied especially to men. The highest proportion of excluded individuals after matching was measured in insured individuals with unknown qualification (26, 35 and 41% combined for men and women for CON1-, CON3- and CON5-subpopulations respectively). This group comprises persons with a foreign degree or those working in temporary jobs, mostly under their factual qualification level.

Differences between men and women are most pronounced for individuals without vocational training and for individuals with unknown qualification, showing a distinctly higher share of excluded individuals in men. In comparison to men, more women were excluded in the group of individuals with vocational training or master craftsman. The differences in the proportion of excluded individuals were moderate for individuals with a university degree. Overall, the exclusions changed the composition of the sample in a different manner for men than for women.

For the third attribute – individual income in terms of salary or pension – a large difference between individuals with and without valid income information was observed. For the CON1-subpopulation only 4% of individuals with valid income information were excluded but 25% of those without known income (summarized for both sexes). These exclusion proportions raise up to 8 and 34% respectively for the CON3- subpopulation and to 12 and 40% for the CON5- subpopulation (summarized for both sexes). Differences between men and women can be noted in particular for the higher income group, where a higher proportion of women was excluded in all three subpopulations.

**Fig. 4:**
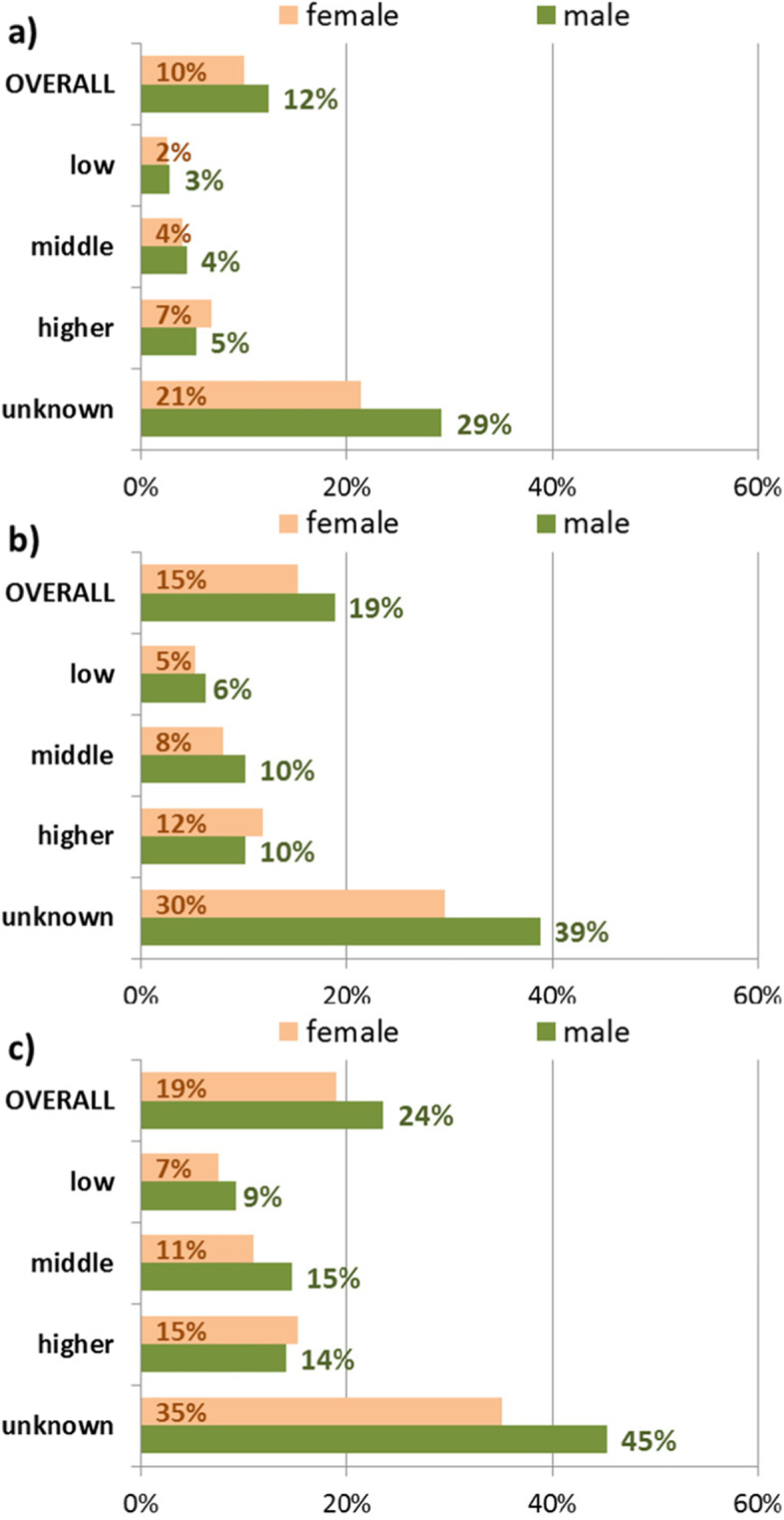
Proportions of excluded individuals by income after application of one (CON1: **a**), three (CON3: **b**) and five (CON5: **c**) years of lookback period, stratified by sex

Persons without reported salary or pension (26% of the sample) showed markedly higher proportions of exclusion, particularly in men. This group consists predominantly of housewives and househusbands, co-insured with their working spouse, unemployed persons, individuals with a minor job under the threshold of social insurance contribution (450€ per month) and other persons, who are insured via the Federal Employment Agency or Social Welfare Office.

### Changes in incidence rates of myocardial infarction and stroke after applying lookback periods

In the following incidence rates of myocardial infarction and stroke are calculated for the four subpopulations showing the effects of a shift in the age structure on crude incidence rates and the effects of selective dropouts by income group on age-standardized rates after applying lookback periods. Crude incidence rates for myocardial infarction and stroke are displayed in Fig. 4, stratified by sex. Changes of the age structure due to the application of lookback periods, as shown above, have effects on the incidence rates. The proportion of younger individuals (under 40 years of age) was reduced due to the application of the lookback periods (Fig. 2). On the other side, the median age of the MI-cases was 70 years (interquartile range 59 to 80 years) and for stroke-cases 77 years (IQR 64 to 83 years). Thus the application of lookback periods affects the denominator far more than the numerator, causing a slight increase of the incidence rates. In case of age-standardization according to the BASE-subpopulation, incidence rates decreased slightly (not displayed).

**Fig. 5:**
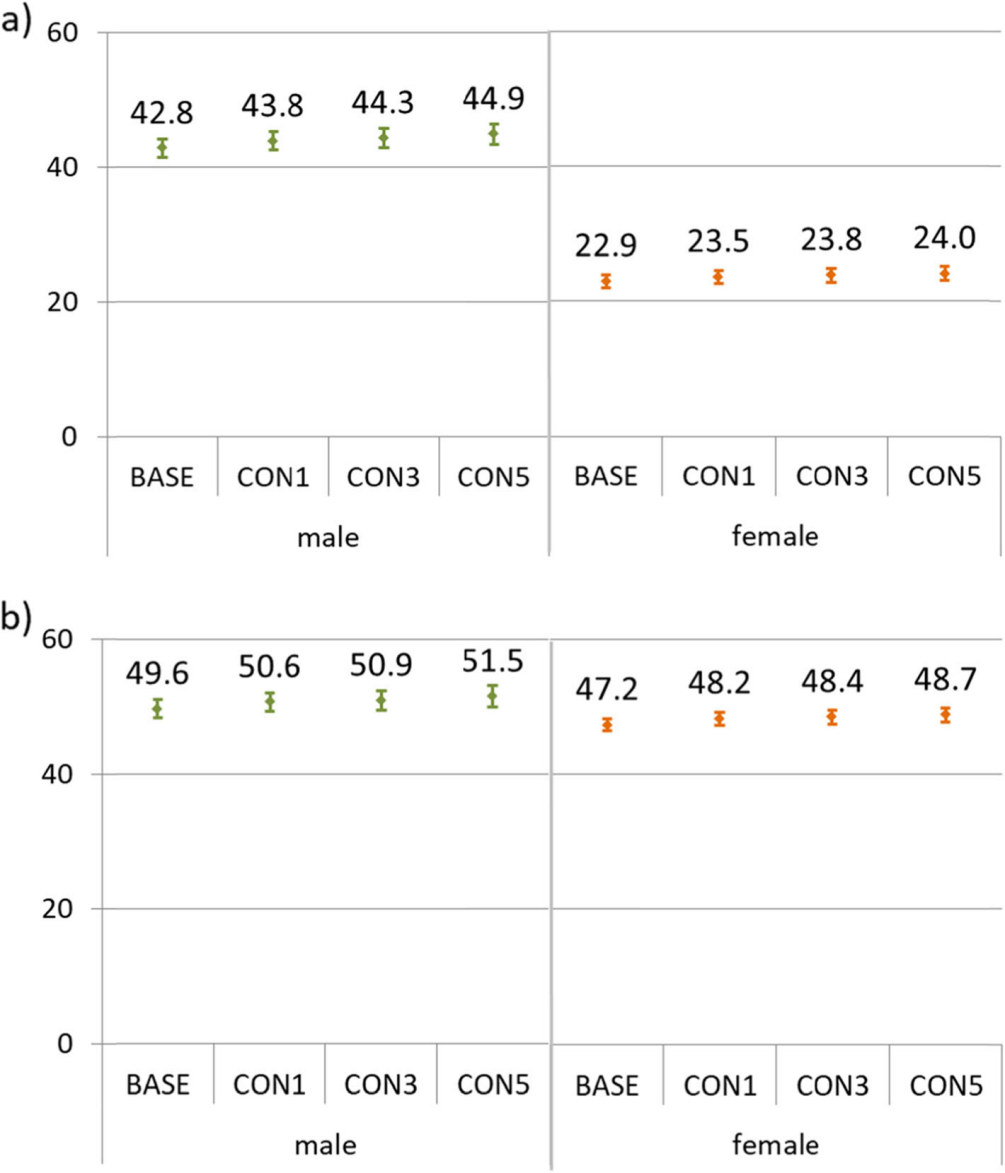
Crude incidence rates of myocardial infarction (upper panel **a**) and stroke (lower panel **b**) in 2017 per 10,000 person-years, stratified by sex, for the subpopulations of 25 years of age and above, according to the application of one (CON1), three (CON3) of five (CON5) years of lookback periods

**Fig. 6:**
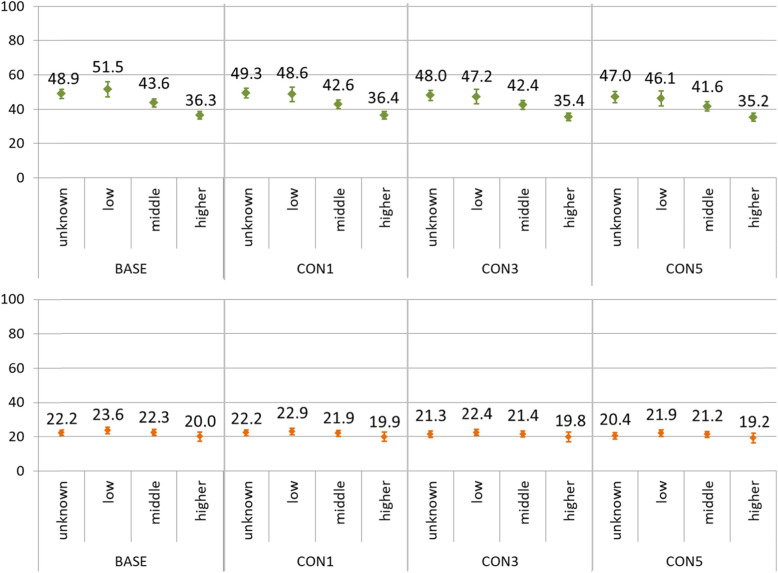
Incidence rates of myocardial infarction in 2017 per 10,000 person-years, stratified by sex and income group, for the subpopulations of 25 years of age and above, according to the application of one (CON1), three (CON3) of five (CON5) years of lookback periods. The upper panel shows incidence rates for men, the lower panel for women. The incidence rates are age-standardized at the BASE-subpopulation

**Fig. 7:**
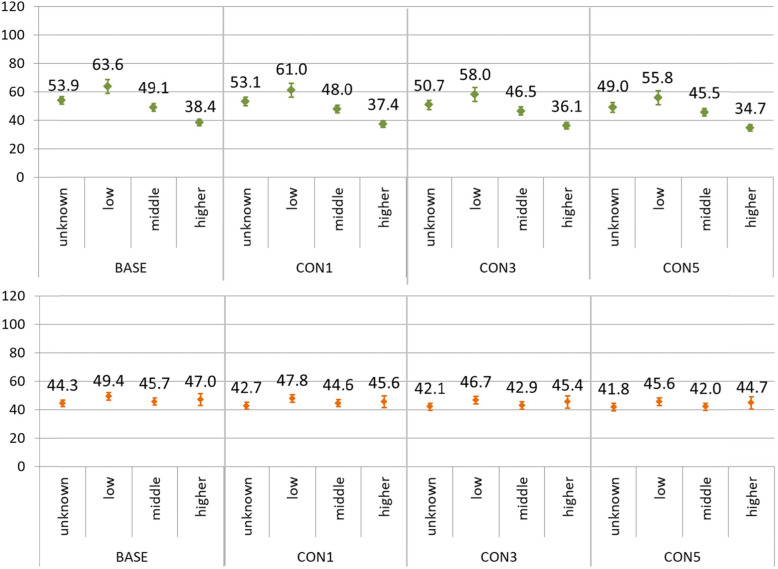
Incidence rates of stroke in 2017 per 10,000 person-years, stratified by sex and income group, for the subpopulations of 25 years of age and above, according to the application of one (CON1), three (CON3) of five (CON5) years of lookback periods. The upper panel shows incidence rates for men, the lower panel for women. The incidence rates are age-standardized at the BASE-subpopulation

Furthermore, depending on the level of qualification or income, proportions of excluded individuals differed after applying lookback periods (Figs. 3 and 5). These differential dropouts have as well an effect on the incidence rates. In Figs. 6 and 7 incidence rates are shown stratified by sex and income group. The calculation was performed with regard to the age structure of the BASE-subpopulation (age-standardized), thus excluding the effect of possible differences in the age structure between the income groups or subpopulations.

For both disease entities, a social gradient in age-standardized incidence rates can be observed in men. For myocardial infarction, a social gradient can also be perceived in women. Interestingly, the difference between the incidence rate for the group with higher income and those with low income diminishes after the application of lookback periods. For example, the difference between low and higher income group amounted to 15.2 cases per 10,000 person-years for myocardial infarction in men in the BASE-subpopulation and shrank to 10.9 cases per 10,000 person-years in the CON5-subpopulationThe development is very similar for stroke (25.2 to 21.1 cases per 10,000 person-years in men). In other words, applying lookback periods leads to a narrowing social gradient in the age-standardized calculation of incidence rates for myocardial infarction and stroke in both sexes.

## Discussion

This study investigated the effects of defining lookback periods used frequently for reducing falsely classified prevalent or recurrent cases as incident. First, changes in the sociodemographic structure were considered. In the second part of the study we showed changes in incidence rates of myocardial infarction and stroke induced by the changing sociodemographic structure after the application of lookback periods.

In the first part, the study focused on changes of the sociodemographic structure of the insurance population of one German SHI fund in 2017 under the condition of continuous insurance for one, three or five years. We observed that proportions of excluded individuals depended on age with younger individuals showing markedly higher dropout rates of up to 43% after applying a lookback period of 5 years. Hence, the age structure of the population changes due to the application of lookback periods leading to a higher proportion of older individuals. Compared to a study population without the precondition of continuous insurance, increased morbidity risks can be expected. This was observed in terms of increasing incidence rates of myocardial infarction and stroke after applying lookback periods (Fig. 4).

With respect to occupational qualification we found differing patterns between men and women with more dropouts in women having completed a vocational training or master craftsman. On the other hand more men were excluded in the group of individuals without vocational training or with unknown qualification. In our data, applying lookback periods leads to an overrepresentation of men with higher qualification and women with lower qualification. Thus, defining lookback periods may result in changes of SES distributions, if lookback periods in longitudinal censored data are applied.

For income, quite low proportions of excluded persons were observed for individuals with valid income information. A specific group with substantial proportions of excluded persons due to the condition of continuous insurance were individuals without known income. Several possible explanations for the higher share of exclusions among this group may apply. On the one hand this group comprises housewives and househusbands, who are co-insured with their employed spouse [23]. In case of a job entry, they have to become paying members of an SHI fund and might decide to change the SHI fund in search for more benefits, as described in the Methods and Data-section. Another group of insured individuals without known income from salaries or pensions are unemployed persons or those with a minor job under the threshold of social insurance contribution (450€ monthly salary), who do not have a paying member of SHI as a spouse (in the latter case they would be co-insured [23]). Unemployed individuals are insured via the Federal Employment Agency or the Social Welfare Office. The case of a job entry can apply here as well, in addition to the case of marriage to a paying member of an SHI fund, followed by a possible change to a co-insured status in another SHI fund. To describe the reverse case – the insured persons with no valid information on income, who are staying within the same SHI fund for at least 5 years, could be housewives and househusbands, who do not change this status due to such reasons as maternal leave or health problems causing inability to work. Elevated morbidity in co-insured individuals was reported for Type 2 diabetes [29]. Another important reason for staying continuously in the same SHI fund with no valid information on income might be long-term unemployment. In contrast the short-term unemployed, who can change between SHI funds more frequently and thus drop out of the population in case of applying lookback periods, the long-term unemployed are insured via Social Welfare Office and would have to stay within one SHI fund until they get a job. As shown in Geyer and Peter [30], long-term unemployed persons have an increased risk of myocardial infarction. Therefore, next to the shift in the age structure towards older ages, selective dropouts in terms of the long-term unemployed might further elevate the morbidity status of an SHI fund after the application of lookback periods.

In the second part of the study we have shown that the application of lookback periods influences incidence rates of myocardial infarction and stroke. Due to the changing sociodemographic structure, especially regarding age, incidence rates increased, leading to an assumption that more impaired persons change their SHI less often. If this assumption applies, studies using lookback periods might overestimate incidence rates in health insurance claims data or similar longitudinal censored data.

Another important finding concerns differential developments of age-standardized incidence rates depending on income. As a consequence, the social gradient observed in men for both diseases and for women for myocardial infarction, decreased after the application of lookback periods, thus raising the question whether social gradients are underestimated in studies using lookback periods.

Selection problems as described here may not only occur in German claims data, but in all databases where individuals may not be represented over the whole life course or over a very long time period. This applies to health care systems where different insurance funds or different insurance systems are coexisting as is the case e.g., in Switzerland [31] or the USA [32, 33], but also in registries where data do not cover the whole population or are censored. In the last decades the use of claims and registry data has increased leading to the increased importance of the topics discussed in this paper.

### Strengths and limitations

This paper is supposed to present an exemplarily analysis of the effects occurring after the application of lookback periods and cannot examine all possibilities. Incidence rates were reported for the whole population and not according to specific age groups in order to demonstrate the overall age effect on incidence rates in this exemplarily analysis of the application of lookback periods. The length of lookback periods was chosen based on the existing literature and out of practicability reasons: there are not many datasets with very long observation times available. Out of the group of diagnoses logically suitable for determining the first occurrence ever, we decided to use these cardiovascular events due to their clarity with respect to the case definition in claims data and known social gradients in the occurrence. Diagnoses occurring in younger ages like diabetes or psychiatric diseases were not used due to methodological complications in the reliable definition of the first occurrence in claims data.

Claims data used in this study are covering a large part of the population in the federal state of Lower Saxony in Germany. Non-response bias is not present, as well as recalling errors in reporting a diagnosis or a date of the disease onset, often existent in survey data [2, 3, 34]. Furthermore, data on impaired persons are included in claims databases, which are often missing in survey data as well [35]. The distinctive feature of these data is the broad range of attributes for sociodemographic analyses. Due to the availability of several SES-measures, collected from employers and pension funds, detailed analyses were possible.

Claims data comprise detailed information on inpatient diagnoses, though only persons with a treated condition of myocardial infarction or stroke can be observed. Individuals who died due to one of the conditions analyzed in this paper before they reached a hospital are not included in claims data. The sociodemographic structure of the population of the SHI fund AOKN is representative for the whole federal state of Lower Saxony and Germany regarding sex and age distribution, though individuals with lower socio-economic status are overrepresented [20]. Due to a higher proportion of individuals with lower socio-economic status in the claims data of AOKN, incidence results in Fig. 4 are probably higher compared to the nationwide level. The overrepresentation of lower SES groups is taken into account as the analyses are stratified by qualification and income.

## Conclusions

The definition of incident cases is often based on disease-free periods in the data. Such preconditions can influence the sociodemographic structure of the population. Especially for German SHI data, researchers should be aware of the selectivities in age structure as well as regarding qualification or income of the study population after the application of lookback periods or due to the condition of continuous insurance. Age standardization could help to encounter the selectivity in age structure, though dropouts of younger persons of up to 50% in the subpopulation of continuously insured persons seems problematic.

A trade-off has to be considered between selectivity effects and eliminating recurrent events for more accuracy in the definition of incidence. The longer lookback periods are, the more precise the definition of an incident case would be, though the structure of the study population would not reflect a typical SHI population any more. SHI claims data or other longitudinal censored data can be used for the estimation of incidence, especially after application of 1 year of lookback. In the case of the application of longer lookback periods, differing composition regarding SES characteristics should be taken into account, e.g. by stratifying or adjusting the analyses.

## Data Availability

The data that support the findings of this study are available from AOKN but restrictions apply to the availability of these data, which were used under special agreement for the current study, and so are not publicly available. Data are however available from the authors upon reasonable request and with permission of AOKN.

## Abbreviations

AOKN: Allgemeine Ortskrankenkasse Niedersachsen (Local Statutory Health Insurance Fund in Lower Saxony, Germany)
CON: Continuously insured persons (definition of three sub-populations in the study)
HIF: Health Insurance Fund
MI: Myocardial infarction
SES: Socio-economic status
SHI: Statutory Health Insurance
